# Mosaic of Anodic Alumina Inherited from Anodizing of Polycrystalline Substrate in Oxalic Acid

**DOI:** 10.3390/nano12244406

**Published:** 2022-12-10

**Authors:** Sergey E. Kushnir, Mikhail E. Kuznetsov, Ilya V. Roslyakov, Nikolay V. Lyskov, Kirill S. Napolskii

**Affiliations:** 1 Department of Chemistry, Lomonosov Moscow State University, Moscow 119991, Russia; 2 Department of Materials Science, Lomonosov Moscow State University, Moscow 119991, Russia; 3 Kurnakov Institute of General and Inorganic Chemistry RAS, Moscow 119991, Russia; 4 Federal Research Center of Problems of Chemical Physics and Medical Chemistry RAS, Chernogolovka, Moscow 142432, Russia; 5 Department of Physics, National Research University “Higher School of Economics”, Moscow 101000, Russia

**Keywords:** anodizing, crystallographic orientation, photonic crystal, anodic aluminium oxide, oxalic acid

## Abstract

The anodizing of aluminium under oscillating conditions is a versatile and reproducible method for the preparation of one-dimensional photonic crystals (PhCs). Many anodizing parameters have been optimised to improve the optical properties of anodic aluminium oxide (AAO) PhCs. However, the influence of the crystallographic orientation of an Al substrate on the characteristics of AAO PhCs has not been considered yet. Here, the effect of Al substrate crystallography on the properties of AAO PhCs is investigated. It is experimentally demonstrated that the cyclic anodizing of coarse-grained aluminium foils produces a mosaic of photonic crystals. The crystallographic orientation of Al grains affects the electrochemical oxidation rate of Al, the growth rate of AAO, and the wavelength position of the photonic band gap.

## 1. Introduction

Porous anodic aluminium oxide (AAO) films possess a unique structure with vertically aligned pores of tuneable diameter and highly controlled interpore distance in the 10–800 nm range. Such a morphology in combination with the high thermal and chemical stability of aluminium oxide makes AAO an extremely popular material for preparing membranes and templates and for other applications in modern materials science and nanotechnology [1,2,3,4].

Previously, it has been shown that the microstructure of an Al substrate influences the anodizing process. In particular, the kinetics of AAO film formation depends on the crystallographic orientation of the Al substrate. The current density is lower for stable Al facets (e.g., (111) or (100)) with dense atom packing [5]. Moreover, the degree of pore ordering is sensitive to the crystallographic orientation of the Al substrate. The smallest fraction of defects (e.g., point defects, high- and low-angle domain boundaries) has been found in AAO formed on Al(100) substrate [5,6,7,8]. The best in-plane orientational pore ordering was observed on grains with (111) orientation [5], whereas the worst ordering was found on Al(110) grains [5,8]. An inclination of the pore growth direction from the normal to the substrate governed by the crystallographic orientation of Al grains has been shown [9,10]. Furthermore, pseudo-epitaxial growth of amorphous AAO with an ordered porous structure within single-crystal grains of Al substrate was discovered [11].

As the AAO film thickness depends on the crystallographic orientation of metal grains [5,12,13], it is logical to assume that an optical path length (the product of the thickness and the effective refractive index) of the AAO should also be dependent on such a parameter. Consequently, a lateral variation in the wavelength position of the photonic band gap (PBG) is expected if polycrystalline Al substrate is used as the starting material for anodizing. However, to the best of our knowledge, there are no reports on the influence of the Al substrate crystallography on PBG. Here, we fill this gap by studying the properties of AAO photonic crystals (PhCs) prepared by the anodizing of Al substrates with mm-sized grains of random crystallographic orientation in 0.3 M H_2_C_2_O_4_.

## 2. Materials and Methods

H_2_C_2_O_4_ × 2H_2_O (99.5%), H_3_PO_4_ (85% aqueous solution), CrO_3_ (99.7%), Br_2_ (98%), and CH_3_OH (99.9%) were used as received, i.e., without further purification steps. All aqueous solutions were prepared with distilled water.

**Figure 1:**
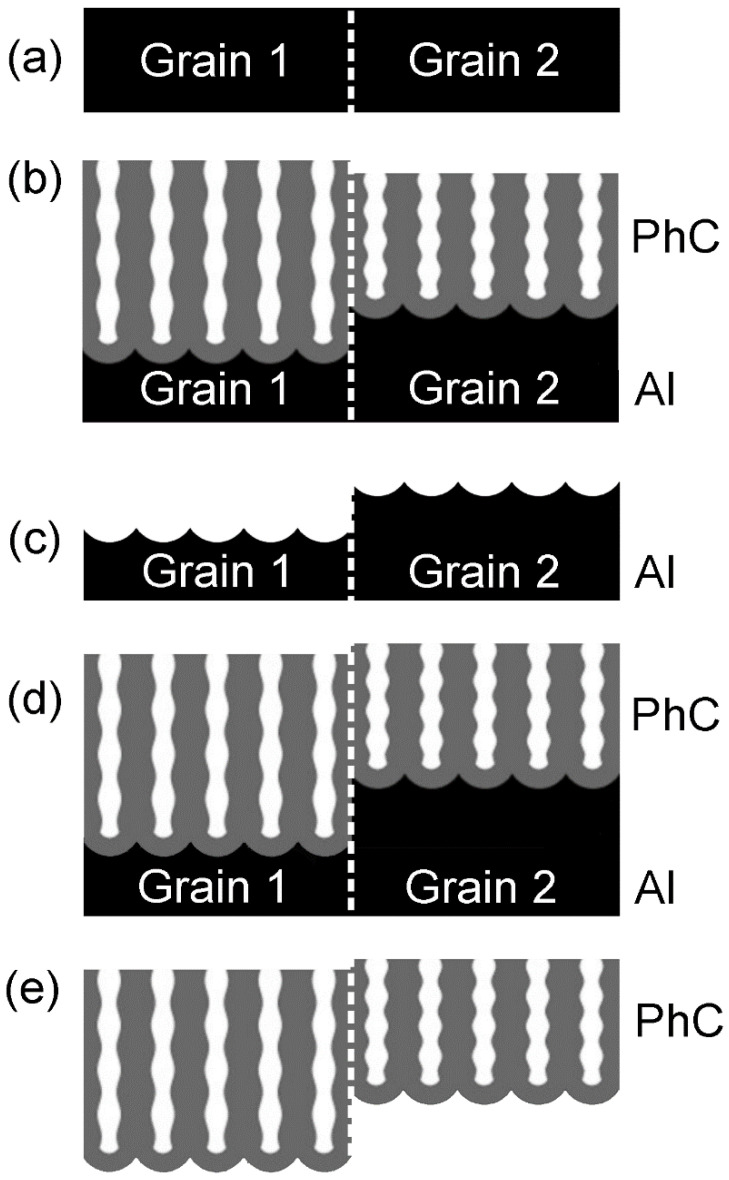
Synthesis of anodic aluminium oxide (AAO) one-dimensional photonic crystal (1D PhC) on coarse-grained Al. (**a**) Mechanically polished Al substrate; (**b**) 1D PhC prepared by Al anodizing; (**c**) Al replica after the selective dissolution of AAO PhC; (**d**) 1D PhC prepared by the second anodizing; (**e**) free-standing 1D PhC after the selective dissolution of Al.

To obtain Al substrates with a coarse-grained structure, high-purity Al foils (99.999%) with a thickness of 0.5 mm were annealed in air using a two-step regime: at 150 °C for 12 h and then at 500 °C for 24 h [14]. A heating rate of 5 °C min^−1^ was used. The surfaces of the recrystallised foils were mechanically polished to a mirror finish (Figure 1a). The microstructure of the Al substrates was examined by electron backscatter diffraction (EBSD) and optical profilometry. The preparation of AAO PhCs was performed on an anodizing area of 0.986–1.250 cm^2^ (Figure S1, Table S1) in a two-electrode electrochemical cell with an Al cathode. The 0.3 M H_2_C_2_O_4_ electrolyte was agitated at a rate of 480 rpm using an overhead stirrer. The electrolyte was maintained at a constant temperature of 0.0 ± 0.1 °C during the anodizing. AAO PhCs were obtained by the anodizing of the Al substrates using voltage (*U*) versus charge (*Q*) modulation, *U*(*Q*) [15,16]:

(1) $$U\left(Q\right)=U_{\mathrm{av}}+2.5\sin(\frac{2\pi Q}{Q_{0}}+\frac{\pi }{2}),$$

where *U*_av_ is the average anodizing voltage, *Q*_0_ *=* 0.53 C is the period of the *U*(*Q*) profile. The number of anodizing cycles was 100 for all the samples, and thus, the anodizing process was stopped when the total charge reached 53 C. The samples were noted as 30–35, 35–40, 40–45, 45–50, 50–55, and 55–60 for the *U*_av_ rates of 32.5, 37.5, 42.5, 47.5, 52.5, and 57.5 V, respectively. After each step of anodizing, the samples were washed repeatedly in deionised water and dried in air. After the first anodizing, the Al replicas (Figure 1c) were obtained by the selective dissolution of the AAO PhCs in a solution containing 0.5 M H_3_PO_4_ and 0.2 M CrO_3_ at 70 °C for 30 min. Prior to the second anodizing under the same conditions as the first (Figure 1d), the topography mapping of the Al replicas was performed. To prepare free-standing 1D PhC (Figure 1e), the Al substrates were selectively dissolved in 10 vol. % bromine solution in methanol.

The grain structure of the Al substrates was examined by EBSD using a NVision 40 scanning electron microscope (Carl Zeiss AG, Oberkochen, Germany) equipped with an Nordlys IIS EBSD detector (Oxford Instruments, Abingdon, UK). EBSD maps were recorded with lateral steps of 20–50 µm.

Measurements of the topography of the Al substrates and free-standing 1D PhCs were performed using a PS50 optical profilometer (Nanovea, Irvine, CA, USA) (lateral step of 5–15 µm). To increase the reflectance of the free-standing 1D PhCs, they were covered with a 50-nm-thick Ag layer using a Q150T ES sputter coater (Quorum Technologies, Laughton, East Sussex, United Kingdom).

The spectral mapping of PhCs was performed in the transmission geometry using a setup based on an ASP-150C optical spectrometer (Avesta Project Ltd., Troitsk, Moscow, Russia). An DH-2000-DUV halogen light bulb (Ocean Optics, Largo, FL, USA) was used as a light source. The light from a 100 µm optical fibre was spatially filtered and focused on the sample (spot size of 0.1 mm, normal incidence, angular aperture of 2.5°). The transmitted light was collected by a 400 µm optical fibre and guided into the spectrum analyser. The sample was mounted on a mechanical XY translation stage to perform mapping with a step of 0.1 mm. The transmittance spectra at various incident angles were measured by rotating the sample around the axis that passed through the light beam in the sample plane.

Analysis of the Fabry–Pérot optical interference fringes was performed by fitting the positions of the extrema observed in the spectra for all measured incident angles. The fitting parameters included two Cauchy dispersion parameters, describing the dispersion of the effective refractive index of anodic oxide [17], the film thickness, and the order of interference of one of the extrema [18,19,20].

## 3. Results and Discussion

**Figure 2:**
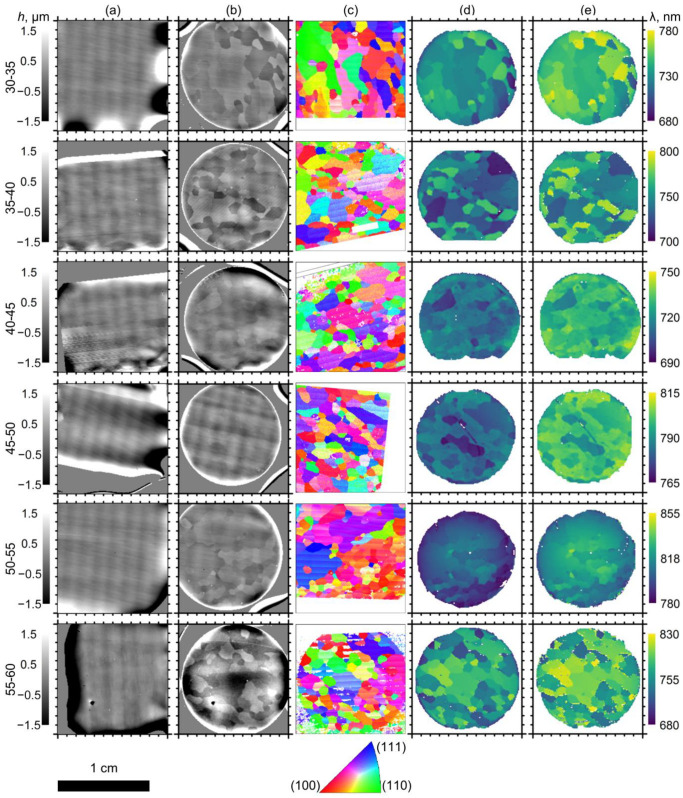
The mosaic of AAO PhCs obtained by anodizing of polycrystalline Al substrates. Height (*h*) maps of the surface of polycrystalline Al substrates before (**a**) and after (**b**) the first anodizing. (**c**) EBSD maps of the surface of polycrystalline Al substrates. The colours refer to the orientations shown by the inverse pole figure for the normal direction. Maps of the central wavelength (λ) of the photonic band gap (PBG) for (**d**) dry and (**e**) wet PhCs. The data in rows correspond to samples 30–35, 35–40, 40–45, 45–50, 50–55, and 55–60. All maps have a lateral size of 12 × 12 mm^2^. White arrows in the panels (b–d) for the sample 30–35 show the region of interest for detailed study (see Figure 3).

Comparing the height maps of the Al surfaces before (Figure 2a) and after (Figure 2b) the first anodizing, it can be seen that the anodizing process uncovered the grain structure of the substrate. According to the EBSD maps (Figure 2c), the anodizing area contained dozens of mm-sized grains (median size of 0.8 mm) with various crystallographic orientations. The shape of the grains on the EBSD maps is identical to the shape of the areas with the same heights, which allows one to perform a correlation analysis of the heights of the Al replica and the Al substrate crystallography. In the case of the samples obtained at anodizing voltages lower than 50 V, the dark grains in Figure 2b with a lower height correspond to the grains close to the (100) orientation (see areas with red colours on the EBSD map, Figure 2c). In contrast, the grains with higher heights (see light areas in Figure 2b) correspond to the grains close to the (111) orientation (areas with blue colours in Figure 2c). Samples 55–60 demonstrate the opposite behaviour: (111)-oriented grains possess lower height and the grains close to the (100) orientation correspond to higher areas on the Al replica (Figure 1c). As the Al was oxidised on all the metal grains simultaneously, obviously the variation in the height of the grains was caused by the variation in the Al electrochemical oxidation rate. In other words, the rate of electrochemical oxidation of Al during anodizing depends on the crystallographic orientation of the Al substrate. For the used 0.3 M H_2_C_2_O_4_ electrolyte, in the 30–50 V range, the fastest rate of electrochemical oxidation was observed for grains close to the (100) orientation, whereas (111)-oriented grains demonstrated the slowest oxidation rate. Anodizing at 55–60 V resulted in the opposite behaviour: (100)-oriented grains became more stable, whereas grains close to the (111) orientation demonstrated the fastest rate of electrochemical oxidation.

The maps of the central wavelength of the PBG (*λ*_PBG_), measured from the same areas as the EBSD maps, are shown in Figure 2d,e for the dry (pores filled by air) and wet (pores filled by water) samples, respectively. The *λ*_PBG_ values varied across the samples (Figure S2). Moreover, the grain mosaics of the spectral and EBSD maps are very similar, which clearly shows the dependence of *λ*_PBG_ on the Al substrate crystallography.

According to the Bragg–Snell law [21], *λ*_PBG_ depends on the period of the PhC structure (*d*), the effective refractive index of the AAO film (*n*_eff_), and the angle of light incidence (*θ*):

(2) $$\lambda_{\mathrm{PBG}}=2d\sqrt{n_{\mathrm{eff}}^{2}-\sin^{2}\theta }.$$

In the case of anodizing in self-ordering regimes at constant voltages, pore deviation from the normal orientation towards the metal surface by a small angle of 1–2 degrees was previously demonstrated [9]. The deviation value is constant within a single-crystal grain, whereas a transition through a grain boundary leads to a sudden change in the pore growth direction. On the other hand, despite various pore inclinations on different crystal grains, the AAO film grew in the normal direction. Thus, this feature of the AAO structure does not affect *θ* in Equation (2) and cannot describe the experimentally observed variation in the PBG position.

In the case of normal incidence, Equation (2) is simplified to:

(3) $$\lambda_{\mathrm{PBG}}=2dn_{\mathrm{eff}}.$$

The experimental *λ*_PBG_ values for the AAO PhC formed on the various Al grains varied with Al crystallography by several percentages for both the dry and wet samples. Furthermore, the wet samples showed a red shift in the *λ*_PBG_ compared to the dry ones. Considering Equation (3), the red shift in the *λ*_PBG_ for the wet samples was caused solely by the increase in the *n*_eff_ because the *d* did not change during the filling of the pores with water. According to the effective medium model [22], the *n*_eff_ is a function of the porosity (*p*), the refractive indices of the AAO cell walls (*n*_cw_), and of a substance filling the pores (air or water). Thus, the ratio (*r*_wd_) of the *λ*_PBG_ values for the wet and dry samples depends on the *p* and *n*_cw_. According to our experiments, the *r*_wd_ was constant over the whole sample area and varied from 1.0196 to 1.0257 for the series of analysed AAO PhCs (Table S2). Taking into account the equality of *n*_cw_ for the entire sample, it can be concluded that the porosity of the AAO film did not depend on the crystallographic orientation of the Al substrate. Consequently, the *n*_eff_ did not depend on the crystallographic orientation of the Al substrate too and the shift in the *λ*_PBG_ from grain to grain was related solely to the variation in the *d*. As the AAO film was formed on all metal grains simultaneously, the variation in the *d* was caused by the variation in the AAO growth rate.

**Figure 3:**
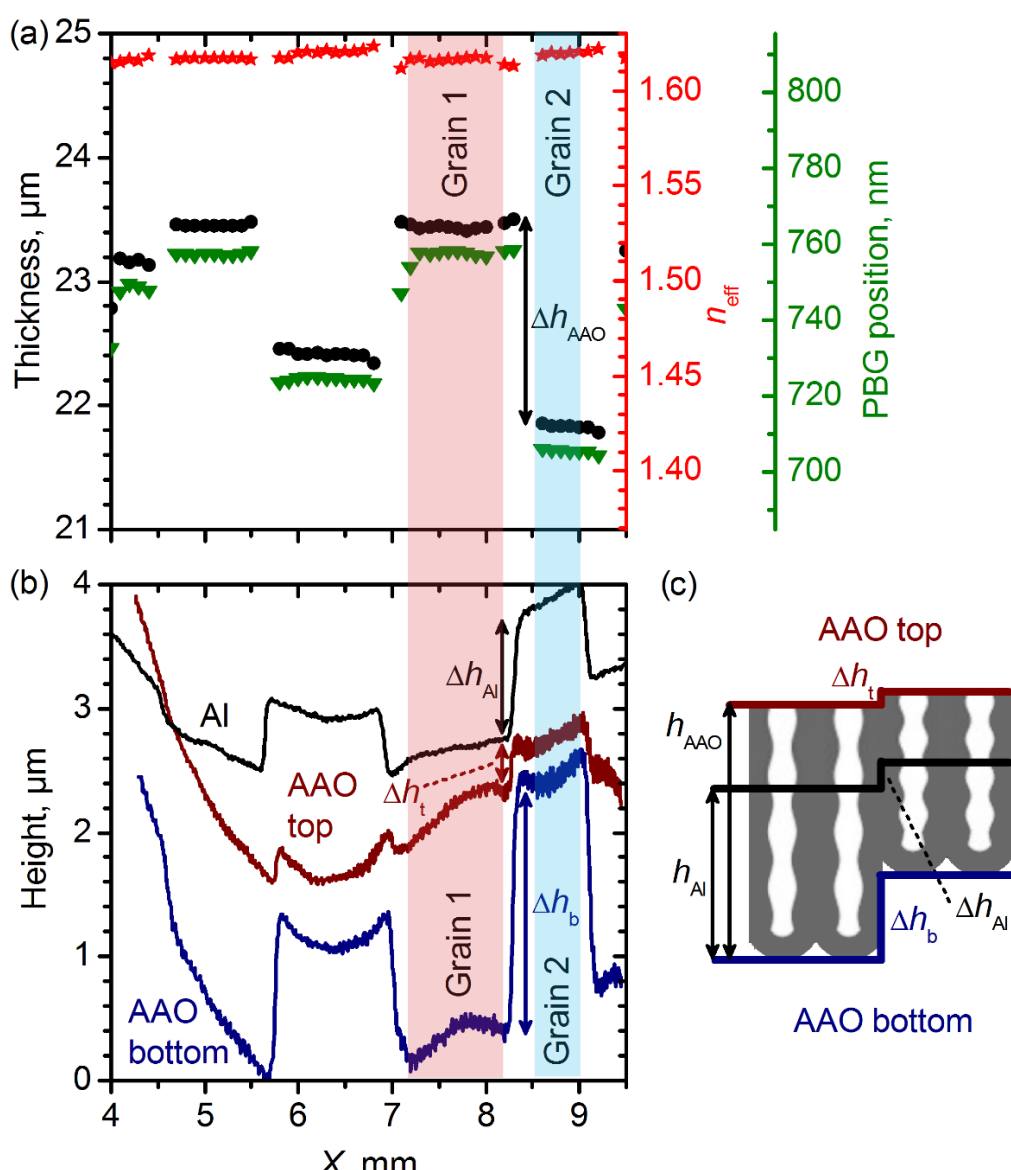
Analysis of profiles measured on the sample obtained at 30–35 V. (**a**) Profiles of thickness (black circles), the effective refractive index at 700 nm (red stars), and *λ*_PBG_ (green triangles). Note that the relative ranges of the vertical axes are equal to 19%. (**b**) Height profiles of the surface of Al substrate after the first anodizing (black) and of the top (red) and the bottom (blue) surfaces of free-standing AAO obtained after the second anodizing. (**c**) Scheme illustrating the places where the height profiles were measured.

Analysis of the Fabry–Pérot optical interference fringes at different incident angles allows one to measure the *n*_eff_ and film thickness (*h*_AAO_) [18,19,20,23]. Figure 3a shows the profiles of *h*_AAO_, *n*_eff_, and *λ*_PBG_, measured along the white arrow (Figure 2, sample 30–35). The strong variation in the *λ*_PBG_ (green triangles) on the grain boundaries correlates with the strong variation in the *h*_AAO_ (black circles), whereas the *n*_eff_ (red stars) is almost constant. Taking account of the equal charge *Q*_0_ = 0.53 C spent for the formation of each structure period and the number of anodizing cycles of 100, one can conclude that *d* = *h*_AAO_/100. Thus, the shift in the *λ*_PBG_ was related solely to the variation in the *d* or the AAO growth rate.

Profilometry of the top and bottom sides of the free-standing 1D PhC was used to measure the height steps on the grain boundaries. Figure 3b shows the height profiles of the surface of the Al substrate after the first anodizing (black) and of the top (red) and bottom (blue) surfaces of the AAO. The profiles were measured along the white line indicated in Figure 2 (see data for sample 30–35). It is worth noting that the samples were not absolutely flat, but a small curvature of the sample surface (ca. 1 µm per mm) did not affect the height steps in the vicinity of grain boundaries. Differences in the electrochemical oxidation rate of Al on grains 1 and 2 caused the appearance of a step on the Al replica with a height of Δ*h*_Al_ after the first anodizing. During the second anodizig, the charge spent for the oxidation was the same, and thus, the step height increased by a factor of 2, since the electrochemical oxidation rates remained the same. As the Al replica reproduced the bottom surface of an AAO film, the height profiles of the Al replica and the AAO bottom were identical. Indeed, the step height at the bottom of the AAO (Δ*h*_b_) was about 2Δ*h*_Al_. The volume expansion during the anodizing [1] and the difference in the electrochemical oxidation rate led to a decrease in the height of the step observed on the top surface of the AAO (Δ*h*_t_) compared to the height difference in the Al replica after the first anodizing Δ*h*_Al_ (Figure 3b). The difference in the thickness of the AAO (Δ*h*_AAO_) formed on grains 1 and 2 was Δ*h*_b_ − Δ*h*_t_ = 1.65 µm. This value is in agreement with the Δ*h*_AAO_ obtained by the analysis of the Fabry–Pérot optical interference fringes (Figure 3a). The profilometry and analysis of the Fabry–Pérot optical interference fringes support the above-mentioned conclusion that the shift in the *λ*_PBG_ from grain to grain was related solely to the variation in the structure periodicity *d*. According to the effective medium model [22], the equality of both the *r*_wd_ (Table S2) and *n*_eff_ (Figure 3a) for the entire sample confirms the equality of both the AAO porosity and the refractive index of the cell walls *n*_cw_, i.e., their independence from the Al crystallography.

**Figure 4:**
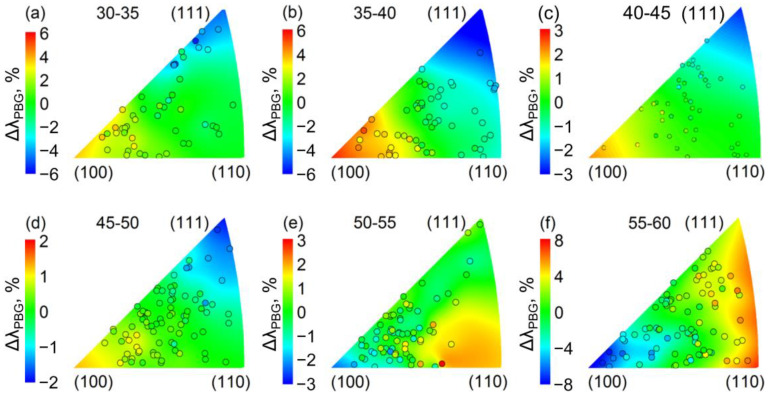
Dependence of the relative shift in the *λ*_PBG_ on the crystallographic orientation of Al substrate in the normal direction for PhCs synthesised at 30–35 V (**a**), 35–40 V (**b**), 40–45 V (**c**), 45–50 V (**d**), 50–55 V (**e**), 55–60 V (**f**). Measurements were performed in air for dry samples. Data points are shown as circles filled with the colour corresponding to the relative deviation from average *λ*_PBG_ and placed on the inverse pole figure in accordance with the crystallographic orientation of the Al grains. The colour map represents the smoothed experimental data for the relative shift of *λ*_PBG_.

The dependence of the relative shift in the *λ*_PBG_ (Δ*λ*_PBG_) on the crystallographic orientation of the Al substrate is summarised in Figure 4. The dependence of Δ*λ*_PBG_ on the crystallographic orientation in the normal direction was similar to the behaviour of the rate of electrochemical oxidation of Al. In the 30–50 V range, the highest Δ*λ*_PBG_ was observed for the PhC formed on the grains close to the (100) orientation, whereas the lowest Δ*λ*_PBG_ was on the Al(111) grains. Anodizing at 55–60 V reversed the dependence: PhCs grown on the grains close to the (100) orientation showed the lowest Δ*λ*_PBG_, whereas the highest Δ*λ*_PBG_ was observed on the grains close to the (111) orientation. As Δ*λ*_PBG_ is determined mainly by the variation in the AAO PhC structure periodicity which is proportional to the AAO growth rate, then the values of Δ*λ*_PBG_ nearly equal to the values of the relative shift in the AAO growth rate.

**Figure 5:**
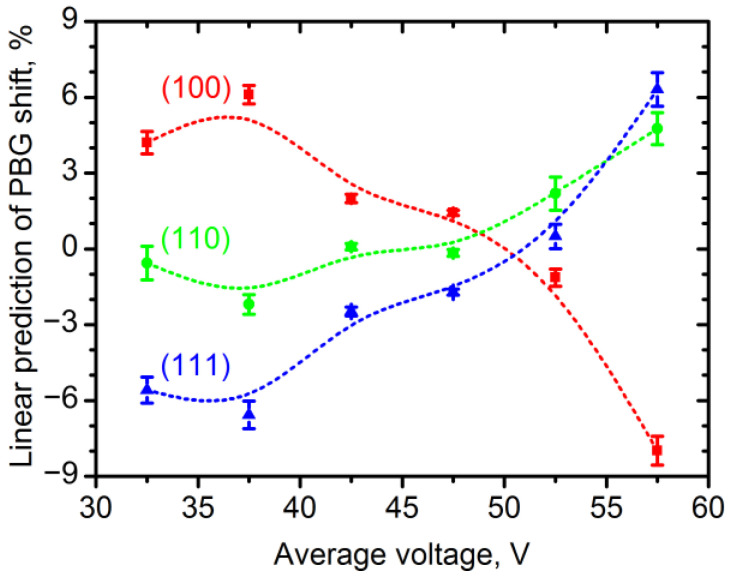
Linear prediction of PBG shift at low index surfaces of Al substrate versus the average voltage used for the synthesis of photonic crystals. The dashed lines are a guide for the eye.

The experimental data on the dependence of the relative shift in the *λ*_PBG_ on the crystallographic orientation were fitted with a plane surface to determine the linear prediction of the relative shift in the *λ*_PBG_ for the low index surfaces of Al (Figure 5). The *U*_av_ in the range of 47.5–52.5 V produced more uniform AAO PhCs, whereas the *U*_av_ of 37.5 and 57.5 V resulted in high values of the relative PBG shift: the *λ*_PBG_ shifted by up to 10 and 14%, respectively, when the crystallographic orientation of the Al substrate shifted from (111) to (100). In the case of anodizing at 40 V in a 0.3 M C_2_H_2_O_4_ electrolyte with and without the addition of 5% of ethanol, Al(100) has demonstrated 15% [12] and 8% [5] higher growth rates, respectively, of the AAO than Al(111). These data are in good agreement with the linear prediction of the relative shift in the *λ*_PBG._

The measured values of the shift in the *λ*_PBG_ are crucial for sensing the application of AAO PhCs [24,25,26,27] where the shift is an analytical response of chemical sensors. In most cases, the shift in the PBG is lower than 1% [24,25,26,27], i.e., the observed effect of the crystallographic orientation of the Al substrate on *λ*_PBG_ is much stronger. Thus, an analytical signal measured from different areas of an AAO PhC sensor synthesised on polycrystalline Al foil with relatively large grains of different crystallographic orientations can lead to wrong results. In photocatalysis applications, the enhancement of the photocatalytic reaction rate by the “slow photon” effect is sensitive to the distance between the edge of the PBG and the absorbance band of the organic molecules [28]. It is expected that the use of PhCs formed on polycrystalline Al substrates can result in a variation in the photocatalytic reaction rate along the sample surface, which then smooths the enhancement effect.

## 4. Conclusions

AAO PhCs with a grained structure were prepared by cyclic anodizing of coarse-grained Al foils in 0.3 M H_2_C_2_O_4_ at 0 °C. The rate of electrochemical oxidation of Al during anodizing depends on the crystallographic orientation of the Al substrate. In the 30–50 V range, the rate of Al electrochemical oxidation increased in the order of Al(111), Al(110), Al(100), whereas the anodizing at 55–60 V reversed the order: Al(100), Al(110), Al(111). A strong correlation between the crystallographic orientation of the Al substrate in the normal direction and the central wavelength of the photonic band gap *λ*_PBG_ was found. The shift in the *λ*_PBG_ was related mainly to the variation in the AAO PhC thickness. The most uniform AAO PhCs were obtained at the average voltage of 47.5–52.5 V, whereas an average voltage of 37.5 and 57.5 V led to high values of the relative PBG shift: the *λ*_PBG_ shifted by up to 10 and 14%, respectively, when the crystallographic orientation of the substrate changed from Al(111) to Al(100). This effect should be considered during the synthesis of high-quality PhC structures and highly sensitive chemical sensors based on AAO. Single-crystalline or highly textured Al substrates with metal grains of similar crystallographic orientation in the normal direction are preferred for the preparation of AAO PhCs if their applications (e.g., sensing, photocatalysis, and optical filtering) require a precisely defined *λ*_PBG_ across the entire sample.

## Data Availability

The data presented in this study are available in the article.

## Supplementary Materials

The following supporting information can be downloaded at: https://www.mdpi.com/article/10.3390/nano12244406/s1, Figure S1: Scanned images of AAO 1D PhCs.; Table S1: Anodizing area (cm^2^) of prepared anodic aluminium oxide one-dimensional photonic crystals; Table S2: The ratio (*r*_wd_) of the *λ*_PBG_ values for wet and dry samples over the entire sample area of prepared anodic aluminium oxide one-dimensional photonic crystals; Figure S2: Transmittance spectra of sample 30–35 measured in the spots grown on grains close to Al(100) and Al(111).
