# Supplementary material for: Mosaic of Anodic Alumina Inherited from Anodizing of Polycrystalline Substrate in Oxalic Acid
Source: Nanomaterials (Basel). 2022 Dec 10;12(24):4406. doi: 10.3390/nano12244406 (PMC9788389; doi:10.3390/nano12244406)
Supplement: Supplementary file 1 [file nanomaterials-12-04406-s001.zip › nanomaterials-2059645-supplementary.pdf]

Supplementary material

Mosaic of Anodic Alumina Inherited from Anodizing of Polycrystalline Substrate in Oxalic Acid

Sergey E. Kushnir [1,2,*], Mikhail E. Kuznetsov [1], Ilya V. Roslyakov [2,3], Nikolay V. Lyskov [4,5] and Kirill S. Napolskii [1,2]

[1] Department of Chemistry, Lomonosov Moscow State University, Moscow 119991, Russia
[2] Department of Materials Science, Lomonosov Moscow State University, Moscow 119991, Russia
[3] Kurnakov Institute of General and Inorganic Chemistry RAS, Moscow 119991, Russia
[4] Federal Research Center of Problems of Chemical Physics and Medical Chemistry RAS, Chernogolovka, Moscow region 142432, Russia
[5] Department of Physics, National Research University "Higher School of Economics", Moscow 101000, Russia
* Correspondence: kushnir@elch.chem.msu.ru

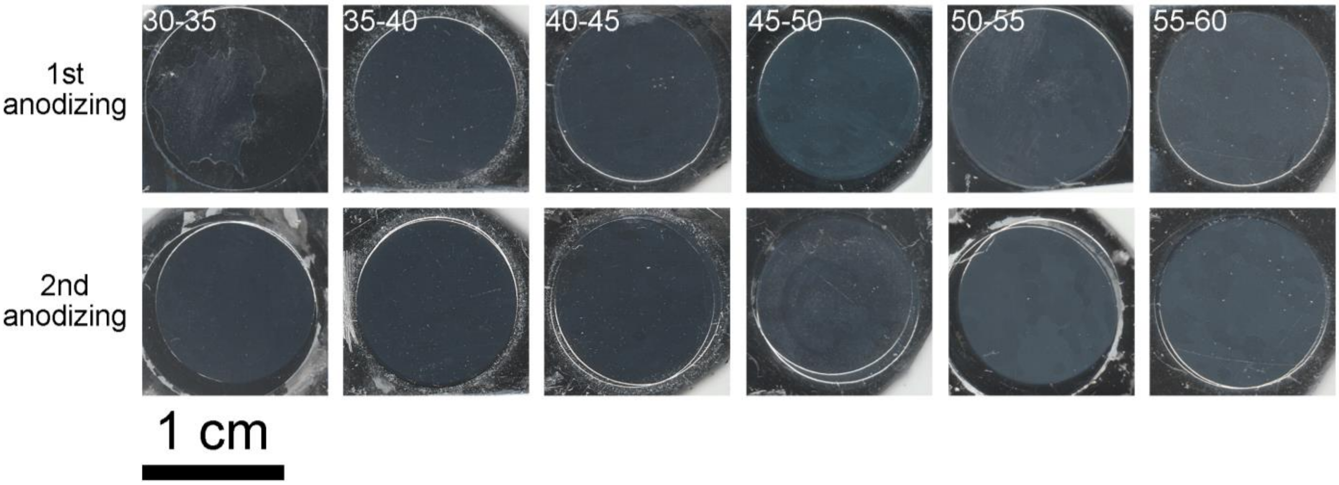

Figure S1. Scanned images of AAO 1D PhCs. The sample names are shown in the upper left corner of the images.

Table S1. Anodizing area (cm^2) of prepared anodic aluminium oxide one-dimensional photonic crystals.

	30-35	35-40	40-45	45-50	50-55	55-60
1st anodizing	1.250	1.063	1.039	1.000	1.213	1.125
2nd anodizing	0.986	1.092	1.088	1.021	1.031	1.100

Table S2. The ratio (r_{wd}) of the λ_{PBG} values for wet and dry samples over the entire sample area of prepared anodic aluminium oxide one-dimensional photonic crystals.

	r_{wd}	standard deviation of r_{wd}
30–35	1.0257	0.0005
35–40	1.0214	0.0005
40–45	1.0224	0.0005
45–50	1.0225	0.0007
50–55	1.0230	0.0003

55–60	1.0196	0.0004

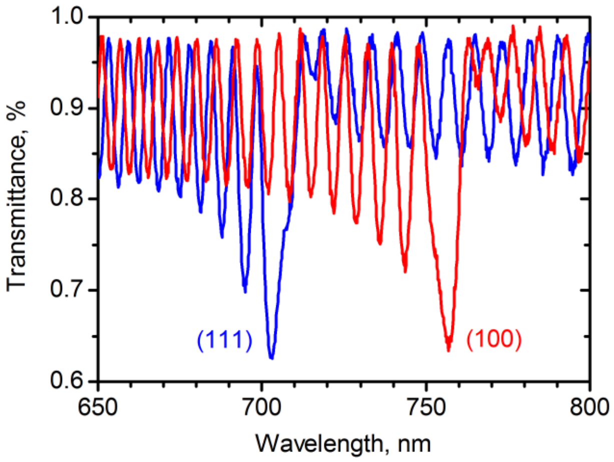

Figure S2. Transmittance spectra of sample 30–35 measured in the spots grown on grains close to Al(100) and Al(111).
